# BioMIPs: molecularly imprinted silk fibroin nanoparticles to recognize the iron regulating hormone hepcidin

**DOI:** 10.1007/s00604-022-05165-0

**Published:** 2022-01-21

**Authors:** Alessandra Maria Bossi, Devid Maniglio

**Affiliations:** 1 Department of Biotechnology, University of Verona, Strada Le Grazie 15, 37134 Verona, Italy; 2 Department of Industrial Engineering, BIOtech Research Center, University of Trento, Via delle Regole 101, 38123 Trento, Italy

**Keywords:** Molecularly imprinted polymers, Silk fibroin, SilMA, Natural biomaterial, Structured peptide, Hepcidin, Competitive fluorescence assay

## Abstract

The possibility to prepare molecularly imprinted nanoparticles from silk fibroin was recently demonstrated starting from methacrylated silk fibroin and choosing a protein as template. Here, we attempted the imprinting of fibroin-based molecularly imprinted polymers (MIPs), called bioMIPs, using as a template hepcidin that is a iron-metabolism regulator-peptide, possessing a hairpin structure. A homogeneous population (PDI < 0.2) of bioMIPs with size ~50 nm was produced. The bioMIPs were selective for the template; the estimated dissociation constant for hepcidin was *K*_D_ = 3.6 ± 0.5 10^−7^ M and the average number of binding sites per bioMIP was equal to 2. The bioMIPs used in a competitive assay for hepcidin in serum showed a detection range of 1.01 10^−7^– 6.82 10^−7^ M and a limit of detection of 3.29 10^−8^ M.

**Graphical abstract:**

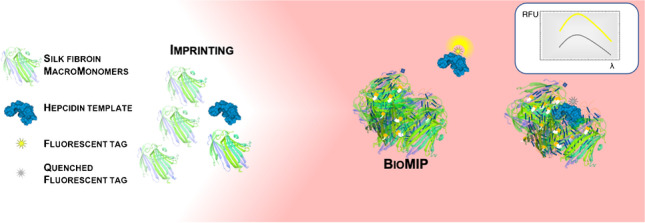

**Supplementary Information:**

The online version contains supplementary material available at 10.1007/s00604-022-05165-0.

## Introduction

Biomimetics prepared by means of a template-assisted synthesis are termed molecularly imprinted polymers (MIPs) [1, 2]. When MIPs are prepared in the form of nanoparticles [3–5], they are referred to either as nanoMIPs or as plastic antibodies [6]. NanoMIPs are synthesized starting from acrylamide-derivative monomers and can eventually be entailed of fluorescence and thermo-responsiveness, so to impart additional functions [3, 7]. Distinctive characteristics of the nanoMIPs are the high affinity and selectivity for their target molecule, rivaling that of monoclonal antibodies, while displaying resistance to high temperatures, solvents, and the possibility to undergo sterilization [8].

NanoMIPs offer advantages and practical solutions for several biomedical applications, such as the replacement of antibodies in assays [9–12], or in cell imaging [13, 14]. Moreover, nanoMIPs can play functional roles in vitro and in vivo; for example, they promote the refolding of polypeptides [15], help the disaggregation of metastatic cell masses [16], contrast G protein–mediated signal transduction in HER-2-positive breast cancer cells [17], or promote the modulation of the fate of stem cells [18]. So far, results are extremely encouraging and nanoMIPs find consideration as proper alternatives to antibodies. Yet, to translate the nanoMIPs into the clinical practice, biocompatibility and non-toxicity are essential requirements. Though encouraging results have been demonstrated for the state-of-the-art nanoMIPs in terms of biocompatibility [19] and non-toxicity [20], the polyacrylamide-based compositions proposed so far for the nanoMIPs might be of concern, particularly for the possibility of specific organ accumulation and for the lack of degradation. In this view, it can be highly desirable to find a route to prepare nanoMIPs from fully natural and biocompatible materials.

Recently, we reported the preparation of silk fibroin imprinted nanoparticles (MIP SF-NPs) [21] starting from the natural biocompatible material silk fibroin (SF) [22]. SF is a protein characterized by a heavy (~390 kDa) and a light chain (~26 kDa) linked together via a single disulfide bond. For the imprinting, we used the heavy chain of SF, extracted from silk cocoon and specifically methacrylated, a material often called SilMA [23]. SilMA possesses pendant double bonds, ideal to be crosslinked during the imprinting process as these can be exploited to stabilize the formed MIP nanoparticle. As a template, to proof the feasibility of imprinting SF-nanoparticles, initially we choose the protein albumin (~66 kDa) [21]. Results indicated that the amount of SilMA in the synthesis solution directly correlated to the size of the formed MIP SF-nanoparticles (50 or 100 nm). The MIP SF-nanoparticles demonstrated selective and specific binding for their targeted protein. Cell viability assays on mouse embryotic fibroblasts showed that the SF MIP nanoparticles were cyto-compatible even at high concentrations (1.5 mg/mL) [21]; thus, the MIP SF-nanoparticles can fully be considered tailor-made functional biological biomimetics, deserving the specific name “bioMIPs.”

Aiming at further exploring the potentials and the limits of the imprinting process of silk fibroin, here we imprinted bioMIPs with a peptide template. Indeed, instead of using a whole and bulky protein, we choose to imprint a structured peptide [15, 24], with the purpose to assess whether the size of the template has impact on the SF imprinting. The selected target analyte was the peptide-hormone hepcidin, which has a molecular weight of 2789 g/mol and is therefore significantly smaller than a whole protein. Hepcidin plays a key role in iron homeostasis [25, 26], being the only regulator of the iron efflux from storage cells to serum [27, 28]; its determination in serum helps the clinical assessment of iron metabolism disorders, offering indications for the prognosis and therapeutic interventions [29, 30]. Hepcidin is a structured peptide, characterized by a hairpin fold tightly, kept in place by four disulfide bridges. Moreover, hepcidin has a marked hydrophobicity and is reported to be highly sticky to lab plasticware; thus, it can be considered a “difficult” analyte for a test [27, 28]. Here, SilMA bioMIPs were imprinted with hepcidin and the formation of imprinted binding sites was verified by isothermal titration calorimetry. At last, the use of bioMIPs to devise an assay for the determination of hepcidin directly in serum samples was explored.

## Experimental

### Materials

SilMA was prepared from silk fibroin from *Bombyx mori* according to the protocol reported in [23]; Hepcidin-25 and fluorescent hepcidin (hepfitc) were from Promega (UK); Lithium phenyl-2,4,6-trimethylbenzoylphosphinate (LAP) was from TCI Chemicals (US); Tris(hydroxymethyl)aminomethane (Tris base), morpholino-ethane sulfonate (MES), sodium dihydrogen phosphate, disodium mono-hydrogen phosphate, sodium chloride, Kalium chloride, human serum albumin, angiotensin, cytochrome c, and serum sample were from Merck Sigma Aldrich (Darmstadt, Germany). All reagents were of commercial grade and used as received.

### Synthesis of bioMIPs

SilMA concentration was adjusted to 0.03% w/v in 10 mM of PBS pH 7.4 buffer, in the presence of 12 μM hepcidin. The photoinitiator LAP was added at the final concentration of 0.2% v/w and photo-polymerized for 10 min with UV light (*λ* = 365nm; 10 W). At the end of the crosslinking process, the print molecule was removed by the addition of Tris free base to the NP suspension to reach a pH of 9.7 for 1 h; then, the MIP SF-NPs were dialyzed (M.W.C.O. 18000 Da) with Milli-Q water 3 × 3 L under mild stirring, followed by dialysis in PBS.

### Dynamic light scattering

Size distribution and polydispersity index (PDI) were determined by dynamic light scattering (DLS) using a Zetasizer Nano ZEN3600 (Malvern Instruments Ltd, Worcestershire, UK) equipped with a 633 nm He-Ne laser. SF-NP samples were dispersed in filtered deionized water at 1 mg/mL or at 0.1 mg/mL. The material refractive index (RI) was 1.490 and the absorption value was 0.01; the dispersant RI was 1.332 for water and 1.340 for PBS, and the viscosity was 0.89 cP for water and 1.02 cP for PBS as reported by the Zetasizer v.6.32 software (Malvern instruments Ltd, Worcestershire, UK). The temperature was set at 298°K and a detection angle of 173° was used. Measurements were collected in triplicate.

### Static light scattering

Static light scattering (SLS) using a Zetasizer Nano ZEN3600 (Malvern Instruments Ltd, Worcestershire, UK) equipped with a 633 nm He-Ne laser was used to measure the number of average molar mass (Mn) of the SF-NPs. Latex monosize standards 15–153 nm (Idc Spheres Portland, UK) were used to calibrate the system. SF-NPs were diluted to five concentrations in the range 5–0.14 mg/mL and measured. Raw data were analyzed by the Debye plot, *KC*/*Rθ* versus particle concentration, where *K* is the optical constant, *C* is the particle concentration, and *Rθ* is the sample Rayleigh ratio; the linear fit intercept corresponds to 1/Mn. A particle refractive index increment (dn/dC) of 0.17 mL/g and a spherical particle shape (Rg = 0.740 Rh) were considered for the estimation of the molecular weight. The RI, viscosity, absorption values, and the Rayleigh ratio were provided by the Zetasizer v.6.32 software (Malvern instruments Ltd, Worcestershire, UK); the refractive index increment (dn/dC) was found in the American Polymer Standards Corporation.

### Scanning electron microscopy

Scanning electron microscopy (SEM) images were obtained using a Supra 40 (Zeiss, Germany) Field-Emission Scanning Electron Microscope (FE-SEM) and a GEMINI column. Images were acquired in secondary electron mode at 5 kV. For the SEM analyses, SF-NPs were suspended in water-ethanol solution at 1 mg/mL; the dispersion was further diluted 10 and 100 times in deionized Milli-Q water. The dispersion was deposited onto a mono-crystalline gold-coated silicon chip (120 nm) and freeze-dried to remove water, preserving tridimensional structure.

### Isothermal titration nanocalorimetry

The Nano ITC Standard Volume (TA Instruments, Newcastle, USA) equipped with a fixed gold cell was used. BioMIP and control SF-NPs were suspended in 50 mM PBS pH 7.4 to the final concentration of 2.5 μM. The titrant, hepcidin, was solvated in PBS to the final concentration of 28 μM. All samples were degassed under vacuum for 5 min. The reference cell was filled with 220 μL of degassed PBS; the sample cell was filled with an equal volume of bioMIP or controls, while 50 μL of titrant was loaded in the syringe. Each ITC experiment consisted of 16 injections of 3 μL at an interval of 300 s from each other. Experiments, performed in triplicate, were conducted at 25 °C. Data were fitted with the independent site model using the Nano Analyze Software v. 3.4.0 (TA Instruments, New Castle, DE), according to manufacturer:

$$\mathrm{Bound}=\frac{\left(-b-\sqrt{b^2-4 ac}\right)}{(2a)}$$

With *K*_a _ value_ = 1/*K*_*D*_; the quadratic constants *a*, *b*, and *c* were defined as follows:

$$a={K}_{\mathrm{a}\_\mathrm{value}}$$

$$b=-{K}_{\mathrm{a}\_\mathrm{value}}\left({\mathrm{MolesSyringe}}_{\mathrm{i}}+n\times {\mathrm{MolesCell}}_{\mathrm{i}}\right)-\left(\frac{{\mathrm{CellVolume}}_{\mathrm{i}}}{10^6}\right)$$

$$c=-{K}_{\mathrm{a}\_\mathrm{value}}\left({\mathrm{MolesSyringe}}_{\mathrm{i}}+n\times {\mathrm{MolesCell}}_{\mathrm{i}}\right)$$

The enthalpy (Δ*H*°) was calculated integrating the heat function:

$$\mathrm{Heat}=\left({\mathrm{Bound}}_{\mathrm{i}}-{\mathrm{Bound}}_{\mathrm{i}-1}\right){10}^9\times \delta h$$

And free energy variation (Δ*G*°) was calculated from *K*_D_ and Δ*H*°.

### Calibration curve for fluorescently labeled hepcidin

For the calibration curve, hepfitc was diluted in the range of concentrations from 1 nM to 6 μM. Measurements were performed on a Tecan Infinite 200 PRO spectrofluorimeter (Tecan, Männedorf, Switzerland) in triplicate on 96 Flat Bottom Black Polystyrene microtiter plates (Thermo Fisher Scientific-Nunclon). Samples were excited at the *λ*_exc_=488 nm and emission was recorded in the range 514–540 nm. Maximum *λ*_em_ was at 522 nm. Linear regression of datapoints: *y* (RFU) = 12.444 × (nM) with *R*^2^ = 0.9979. Additionally, the linear regression for RFU as function of the quantity of hepfitc (pmol) was as follows: *y* (RFU) = 60.427 × (pmol) with *R*^2^ = 0.9997.

### Binding isotherms in fluorescence

Measurements of the bioMIP’s binding abilities were performed using hepfitc as target analyte. Measurements were performed in triplicate on 96 Flat Bottom Black Polystyrene microtiter plates (Thermo Fisher Scientific-Nunclon). Wells were loaded with 37.5 μg of bioMIPs and incubated with increasing concentrations of hepfitc (3 nM–2.5 μM) in PBS 10 mM pH 7.4 supplemented with 0.08% Tween-20. Control experiments were performed in the same conditions but with non-imprinted SF-NPs. The emitted fluorescences for hepfitc (3 nM–2.5 μM) in PB 10 mM pH 7.4 supplemented with 0.08% Tween-20 incubated with the presence of bioMIPs or of SF-NPs were taken at 30 min incubation with a Tecan Infinite 200 PRO spectrofluorimetric microplate reader (Tecan, Männedorf, Switzerland), setting the excitation at *λ*_exc_ = 488 nm and recording *λ*_em_ = 522 nm. Data were transformed in bound quantity (pmol) by using the calibration curve reported in the section “Calibration curve for fluorescently labeled hepcidin.”

The dose-response curve was fitted by the Langmuir equation model here recalled:

1 $$\left|\mathrm{bound}\right|=\left|\mathrm{bound}\_\max \right|\left(\frac{c}{K+c}\right)$$

where *c* is the analyte concentration, and bound_max is the maximum value of binding, calculated by the saturation value minus the blank value.

### Selectivity of the bioMIPs

The selectivity of bioMIPs for the template and for non-related peptides and proteins, was tested as follows: bioMIPs were incubated in the presence of hepfitc (500 pmol) alone, or in the presence of one of the following competitors: hepcidin (100 nmol); the sequence non-related peptide angiotensin (6 nmol); a non-related protein cytochrome c (6 nmol); a non-related serum abundant protein human serum albumin (6 nmol). Fluorescence was measured as explained above; the measurements were performed in triplicate. Quantities of bound hepfitc were plotted as function of the competitor, by using the calibration curve.

### BioMIP competition in serum

Commercial pooled serum from healthy donors (Sigma-Aldrich) was depleted of hepcidin prior to use. The assay was performed in triplicate on black 96 flat bottom–well microtiter plates (Thermo Fisher Scientific-Nunclon). Wells were loaded with 30 μg/well of bioMIPs and incubated with serum (10 μL) in a final volume of 150 μL PBS 10 mM pH 7.4 supplemented with 0.08% Tween-20 in the presence of hepfitc (500 pmol or 250 pmol). Competition was performed in a same condition but with the addition of increasing quantities of hepcidin competitor to the wells (0.11, 0.34, 0.68, 2.70 μM). Fluorescent measurements were takes as above. Data were plotted as the percentage of competition and were fitted with the Hill model equation, so to determine the ligand affinity and to roughly estimate the number of binding sites per bioMIP:

2 $${y}_{\mathrm{x}}=\left|{y}_{\mathrm{min}}\right|+\left(\left|{y}_{\mathrm{max}}\right|-\left|{y}_{\mathrm{min}}\right|\right)\times \frac{x^n}{k^n+{x}^n}$$

where *x* is the analyte concentration, *y*_x_ is the percentage of displacement at the concentration *x*, *y*_min_ is the maximum % of displacement, *y*_max_ is the maximum % of displacement, *k* is the dissociation constant, and *n* is the index of cooperativity, that can be taken as an indication of the number of available binding sites.

## Results and discussion

### Preparation and physical characterization of the bioMIPs

The preparation of the imprinted, silk fibroin–based nanoparticles, called bioMIPs, was adapted from the protocol published earlier [21]. Briefly, the starting material, methacrylated SF or SilMA [23], was diluted in aqueous conditions (PBS, final *V* = 4 mL) to the concentration of 0.03% w/v [21]. It is expected that, due to these highly diluted conditions, the SF proteins that spontaneously form supramolecular aggregates are forced to aggregate in a limited number of units, ultimately yielding to the formation of SF-nanoparticles (SF-NPs). When the template is placed in the solution, statistically, some of the forming SF-NPs will organize around the template, yielding to the formation of bioMIPs. To stabilize the bioMIPs, we selected the photo-crosslinker lithium phenyl-2,4,6-trimethylbenzoyl phosphinate (LAP, 0.2% w/v) that is commonly used in the preparation of SilMA scaffolds [23, 30]. Irradiation with UV light for 10 min ensures the crosslinking of the bioMIPs. At the completion of the polymerization, bioMIPs are incubated at high pH, so to negatively charge both the template and the bioMIPs, favoring the removal of the template, followed by extensive dialysis. Finally, the bioMIPs are equilibrated in PBS and used or stored at 4 °C for up to 48 h.

**Fig. 1:**
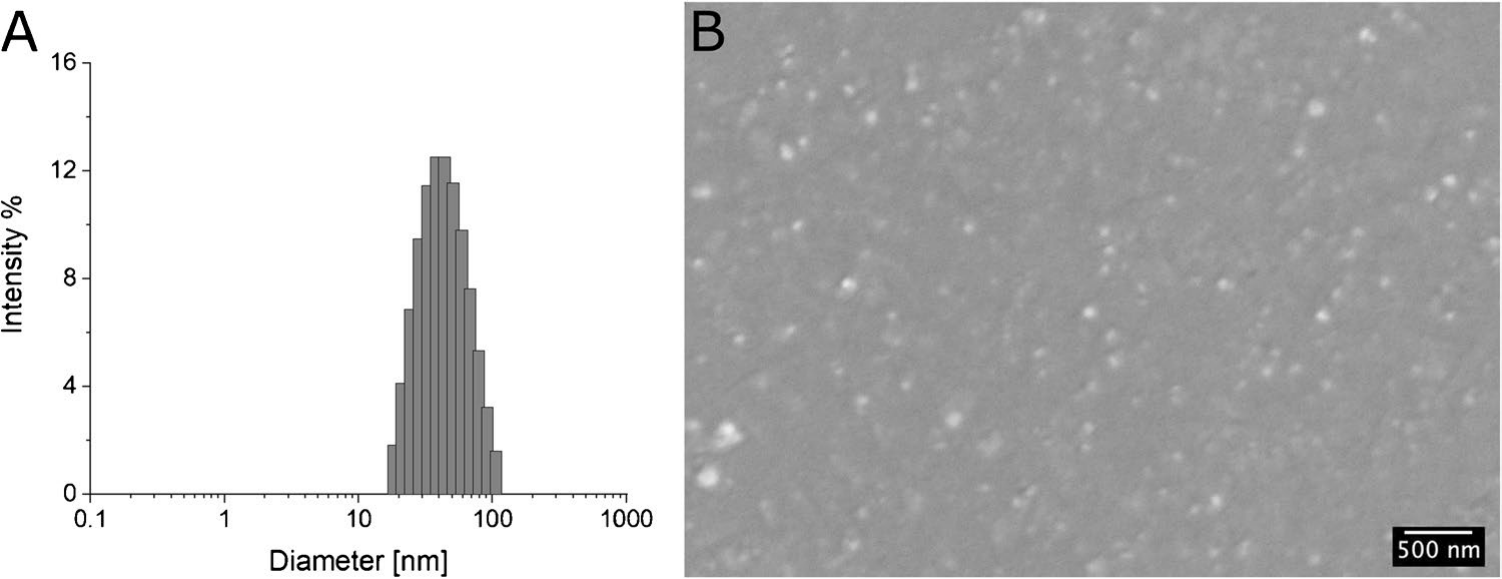
BioMIP size distribution obtained by DLS (**A**) and micrograph obtained by SEM after freeze-dried on top of a silicon slide (**B**)

The size of the bioMIPs, determined by dynamic light scattering (DLS; Fig. 1A), showed an averaged hydrodynamic size (*Z*_ave_) of 46 ± 5 nm and a polydispersity index (PDI) of 0.18, suggesting the process forms homogeneous nanomaterial. Scanning electron microscopy (SEM) confirmed the averaged dimensions of the NP population (Fig. 1B). The estimated molecular weight for the bioMIPs was 1.4 ± 0.3 10^6^ Da (Fig. SI 1).

### Affinity and specificity of the bioMIPs

**Fig. 2:**
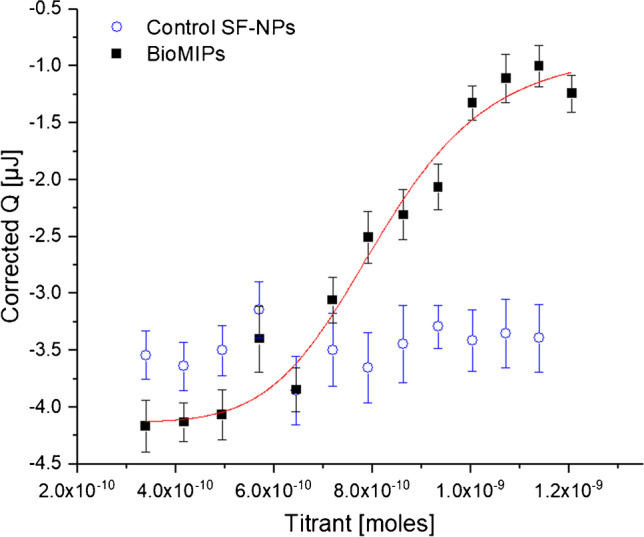
Isothermal titration calorimetry. Hepcidin titrated to control SF-NPs (blue open circles) showed non-specificity; in contrast, hepcidin titrated to bioMIPs (solid squares) showed a saturation course that was fitted an independent binding site model (red line). Fitting parameters are reported in Table 1. Data are the mean value of triplicate

The formation of imprinted binding sites on the bioMIPs was proven by isothermal titration calorimetry (ITC). BioMIPs (2.5 μM, *V* = 220 μL) and control SF-NPs (NIP, 2.5 μM, *V* = 220 μL) were respectively titrated with hepcidin (28 μM, *V* = 50 μL). The interaction between the peptide and the NPs during the titration process resulted in heat exchanges (Fig. 2). In particular, the titration of the bioMIPs with the template showed a saturation profile (Fig. 2, solid squares), whereas no clear trend was observed for the control material (Fig. 2, open circles), suggesting, in the latter case, non-specific interactions. The analysis of the thermograms allowed estimating the binding constant and the thermodynamic parameters for the bioMIP/hepcidin pair [31–33] that are reported in Table 1. The hepcidin/bioMIP interaction confirmed to be a spontaneous enthalpy-driven process, having a negative Gibbs free energy variation. The estimated dissociation constant (*K*_D_) was 3.6 ± 0.5 10^−7^ M. The average number of binding sites per particle (*n*) was 2. Overall, these results demonstrated the success of the imprinting of a structured peptide in SilMA-based bioMIPs, thus proving the imprint of peptide templates is feasible with SilMA macromolecular monomers. Moreover, from Table 1, we can infer initial, despite incomplete, rules for the bioMIP’s imprinting process. It seems that, when the chosen template is a peptide (hepcidin, 2789 g/mol, estimated hydrodynamic diameter 0.7 nm), the affinity for the target analyte is lower (Table 1, dissociation constant *K*_D_ value), than when the template was a protein (albumin, 66000 g/mol; estimated hydrodynamic diameter 6 nm [21]). Data suggest a positive correlation: the larger the template, the higher the affinity of the imprinted binding site on the bioMIP. Instead, the averaged number of binding sites per bioMIP appears to be greater for the imprinting of a peptide, in respect when a protein is imprinted, indicating an inverse correlation between the number of imprinted binding sites and the size of the template.

**Table 1:** Thermodynamic parameters of the nanocalorimetric titration for the pair bioMIP/hepcidin and for the pair albumin-imprinted SF-NPs/albumin; data fitted assuming independent binding sites and thus via an independent binding site model equation (“Experimental”)

Name	*K*_D_	*n*	Δ*H*°	Δ*G*°	Reference
	M		kJ/mol	kJ/mol	
BioMIP	3.6 ± 0.5 10^−7^	2.10 ± 0.25	−58.5 ± 7.8	−36.4 ± 8.1	This work
Albumin-MIP SF-NPs	5.7 ± 0.3 10^−8^	1.25 ± 0.54	-	-	[21]

**Fig. 3:**
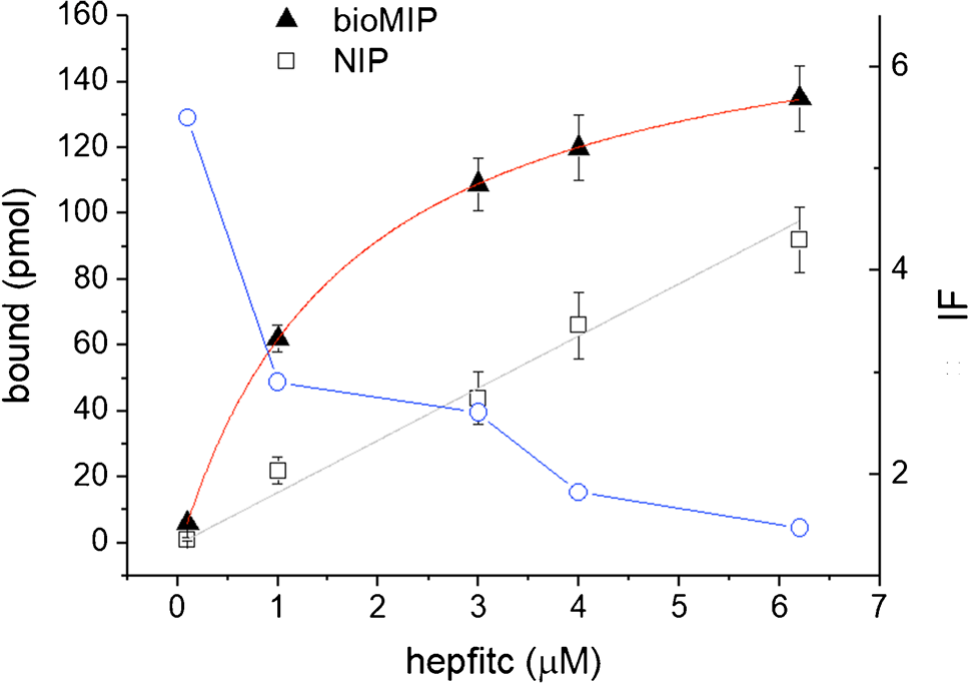
Fluorescently tagged hepcidin, hepfitc, binding to bioMIPs (solid triangles) compared to hepfitc binding on control nanoparticles (NIP, open squares). Hepfitc bound to bioMIPs showing a saturation course; the Langmuir fitting model is reported as red line; the *K*_Dapp_ = 1.67 μM. The imprinting factor (IF, blue open circles) that is expressed as the ratio between the quantity of hepfitc bound to bioMIP with respect to NIP was within the value 2.5, for the hepfitc concentration ≤ 3 μM. Lower IFs were observed for hepcidin >3 μM: the drop in IF is attributed to the increment in non-specific adsorption, given the high hydrophobicity and stickiness of the analyte

Fluorescence spectroscopy was employed to gain an independent confirmation of the binding specificity of the bioMIPs. In this case, a fluorescently labeled hepcidin (hepfitc) was used. Prior to start, a hepfitc (*λ*_ex_ 495 nm, *λ*_em_ 522 nm) calibration curve was prepared (Fig. SI 2, equation in “Experimental”). As reported in Fig. 3, the binding of hepfitc to bioMIPs shows a saturation profile (solid triangles). The bioMIP binding dataset was fitted with the Langmuir equation model, to estimate the apparent dissociation constant value in fluorescence, which was *K*_Dapp_ = 1.67 μM. Moreover, the hepcidin binding to control NIP nanoparticles showed a typical non-specific linear trend (Fig. 3, open squares). The interaction strength of the imprinted polymer towards the template is measured by the parameter called imprinting factor (IF) [34]. The IF (Fig. 3, blue open circles), calculated as the quantity of hepfitc bound to the bioMIP with respect to that bound to the NIP, was >2, for the concentrations of hepfitc ≤ 3 μM. IF diminished when hepfitc concentration raised above 3 μM, possibly as a consequence of the increased quantity of hepcidin non-specifically adsorbed to the NIP [26]. A way to minimize adsorption is the use of buffer supplemented by elevated quantities of non-ionic or zwitterionic surfactants [35], which in turn has the counter-effect to decrease the bioMIP/template specific interaction; thus, a compromise condition had to be chosen.

### Selectivity of the bioMIPs

**Fig. 4:**
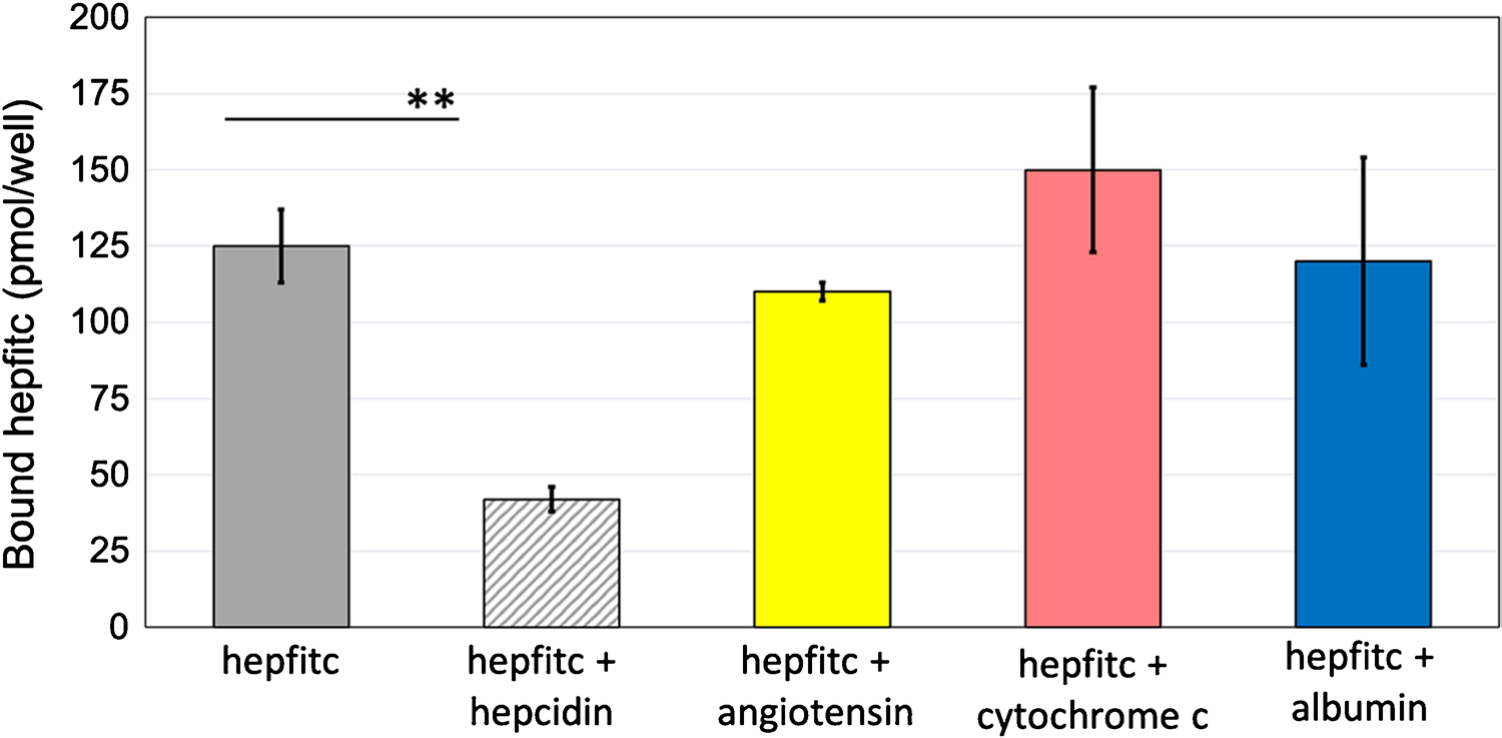
Selectivity of the bioMIP binding evaluated by a competitive assay. The displacement of bound hepfitc (500 pmol/well) was observed when the competition was hepcidin (1.8 nmol/well, gray stripes bar), whereas a non-related peptide (angiotensin, 6 nmol/well, yellow bar) or proteins (cytochrome c, 6 nmol/well, pink bar; albumin, 6 nmol/well, blue bar) did not compete for the imprinted binding sites. Data were collected at least in triplicate. **p < 0.05

The selectivity of the bioMIPs was assessed by means of a competitive binding assay, based on the fluorescently labeled hepcidin (hepfitc) in the presence of different competitors. The results (Fig. 4) showed that in the chosen assay conditions the hepfitc bound at 30 min was 125 ± 18 pmol (solid gray bar). When hepcidin was used as a competitor, a significant displacement of the bound hepfitc (striped gray) was observed (*p* < 0.05). In contrast, when the biopeptide angiotensin, which has an amino acid sequence not related to hepcidin, was chosen as competitor, no influence on the bioMIP/hepcidin binding was observed. Seemingly, both the proteins cytochrome c and albumin provided no displacement of the bound hepfitc. These results clearly confirmed the selectivity of the bioMIPs for their target molecule.

### BioMIPs for the determination of hepcidin

**Fig. 5:**
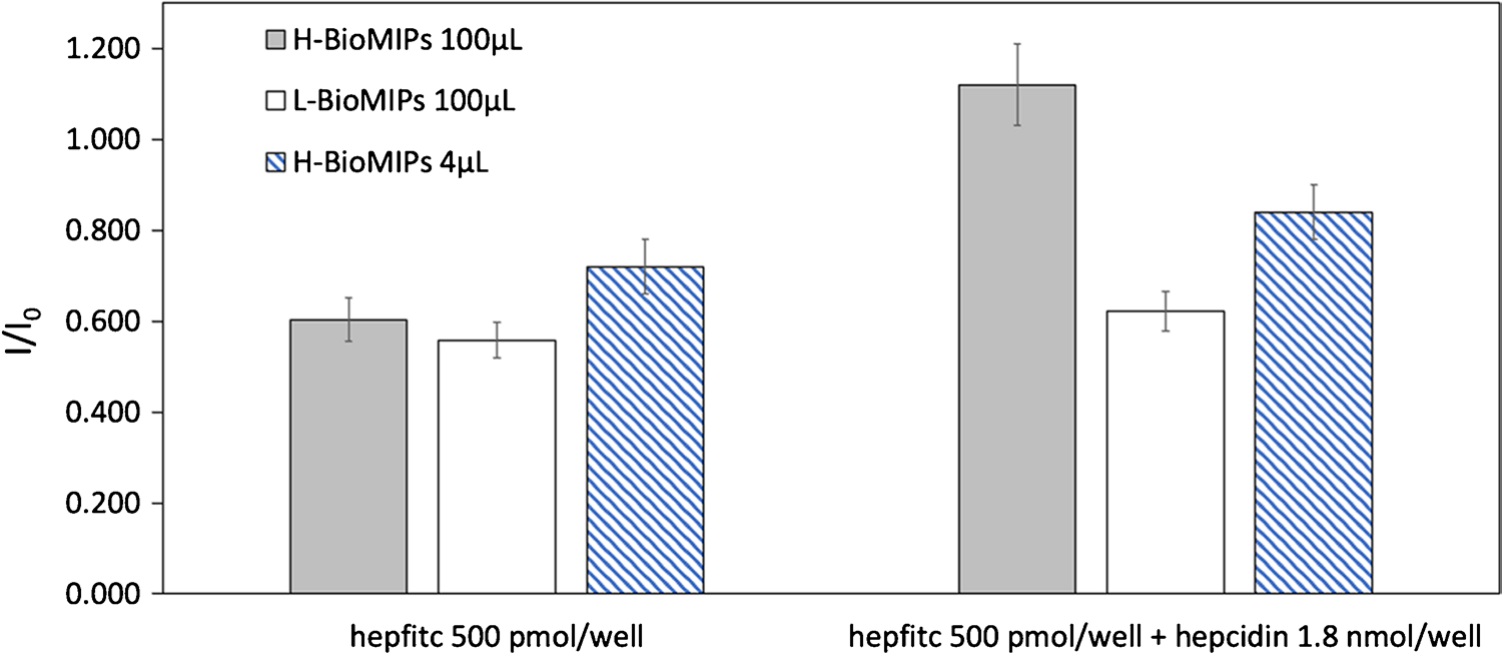
Relationship between the quantity of the bioMIP’s binding sites and the displacement. Tested conditions were as follows: 100 μL of H-BioMIPs (gray bars); 100 μL of L-BioMIPs (white bars); 4 μL of H-BioMIPs (blue stripes bars). The competition was in PBS-Tween 0.08% v/v

Proven that the hepcidin-imprinted bioMIPs showed both selectivity and affinity for the template, we attempted to apply the bioMIPs to the determination of hepcidin. To define the best conditions for the measure, the bioMIPs were imprinted with different quantities of template. BioMIPs were prepared in the presence of either a high template quantity (hepcidin 1 μmol, *V*_tot_ = 4 mL; H-BioMIPs) or with low template (hepcidin 107 nmol, *V*_tot_ = 4 mL; L-BioMIPs). To assess the most suitable imprinting condition for the measure, both kinds of bioMIPs were tested for the displacement of hepfitc (500 pmol/well) by the competitor hepcidin (1.8 nmol/well) at the set time of 30 min of incubation. BioMIPs (either 4 μL or 100 μL of H-BioMIPs, or 100 μL of L-BioMIPs) were incubated in the presence of hepfitc and of the competitor hepcidin for 30 min and then measured. According to the Stern-Volmer equation, the emitted fluorescence of hepfitc in the presence of bioMIPs was used as reference value *I*_0_, whereas the fluorescence emitted in the presence of the competitor was referred to as *I*. The results, reported in Fig. 5, showed a clear displacement effect for the competition (hepcidin/hepfitc) taking place on H-BioMIPs 100 μL (gray bars; I/I_0_ = 1). When L-BioMIPs 100 μL were tested, these did not show competition at 30 min (white open bars). We hypothesized that the number of imprinted and accessible binding sites on L-BioMIPs was not adequate for the chosen measurement conditions. The value of *I*/*I*_0_ < 1 is possibly given by non-specific adsorption of hepfitc to L-BioMIPs. Upon hepcidin addition, the non-specific adsorption is not perturbed at the chosen incubation time. As a control, we checked the competition effect on H-BioMIPs, this time used at low concentration 4 μL, so to reduce 20 times the number of binding sites in the test (blue stripes bars). As a result, we observed slight tendency to displacement, though insufficient (*I*/*I*_0_ < 1), which find interpretation in the lack of a suitable quantity of binding sites for the measure.

### BioMIPs to determine hepcidin in serum

**Fig. 6:**
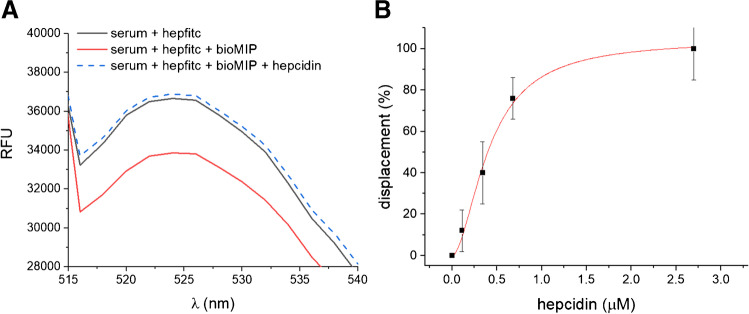
Competition assay in serum. **A** Fluorescence emission spectra of hepfitc in a hepcidin-depleted serum (black line, *I*_0_). The addition of bioMIPs to hepfitc serum produces quenching (red line, *I* < *I*_0_), that is, the formation of the complex bioMIP/hepfitc. Spiking non-labeled hepcidin (2.7 μM) to bioMIP/hepfitc serum (dotted blue line) had a competition effect (*I* = *I*_0_). **B** Competitive assay for the determination of hepcidin in serum; the dynamic range of response and the assay parameters are reported in Table 3.

With the purpose to apply the bioMIPs for the hepcidin detection in biological samples, we translated the bioMIP competition conditions from PBS to serum. For this, hepcidin-depleted and pooled serum samples were used at a 20-fold dilution. Serum samples were spiked with hepcidin (3 μM). The preliminary test to assess the feasibility of the competition in serum is reported in Fig. 6A. The fluorescence of diluted serum samples spiked with hepfitc (500 pmol/well) is reported in Fig. 6A as a black line (*I*_0_). The emission (*I*) quenching measured for bioMIPs (30 μg) co-incubated with hepfitc-spiked serum was considered the maximal binding in the assay conditions (Fig. 6A, red line). Next, bioMIPs were incubated with hepfitc-spiked serum together with an excess of non-labeled hepcidin (1.8 nmol/well). In such conditions, hepcidin competed with hepfitc and the measured fluorescence reverted to the starting level *I*_0_, indicating full hepfitc displacement (Fig. 6A, dotted blue line). These results proved that the competition takes place also in serum samples. However, the displacement was not observed at early times (30 min), but from ≥ 120 min of incubation. Such a difference with respect to the analytes in PBS finds explanation being the complexity of the serum composition. It has been proven that long incubation times are needed for the thermodynamical reorganization of the millions of proteins composing serum on affinity tags [36–38], this most likely applying also to the bioMIP binding sites.

The dynamic range of response for the bioMIP measurement of hepcidin in serum was explored in competition, using hepfitc at 250 pmol/well. In the case, hepcidin was spiked in sera at 0.11, 0.34, 0.68, and 2.70 μM. Sera were incubated in the presence of hepfitc and the emitted fluorescence was measured. The results, shown in Fig. 6B, indicate displacement in the tested range of concentrations, thus validating the original use of bioMIPs to detect hepcidin directly in serum. Data of the displacement isotherm were fitted with a Hill model equation and are reported in Table 3. Hill equation was chosen because it estimates both the dissociation constant (*K*_D_ = 4.10 10^−7^ M) and the *n* parameter (*n* = 1.79) that is the indicator of multimeric receptorial sites. Because bioMIPs are synthesized in solution, it is expected that the imprinted binding sites would be heterogeneous. Indeed, this assumption was verified by nanocalorimetry (Table 1) where the number of binding sites per bioMIP was estimated as equal to two and found further confirmation in the serum competitive assay (Table 2). Finally, the analytical parameters for the competitive assay are reported in Table 3, showing the assay is linear in the high nanomolar range and has a limit of detection (LOD) equal to 3.29 × 10^−8^ M.

**Table 2:** Parameters for the bioMIP displacement isotherm for hepcidin in serum

Name	*B*max	*n*	*K*_D_	Statistics
	Value	Value	*M*	Adj *R*-square
bioMIP	104.26 ± 4.70	1.79 ± 0.26	4.10 10^−7^ ± 0.04 10^−7^	0.993

**Table 3:** Parameters for the competitive bioMIP assay

Parameters	Value
*K*_aff_ [*M*^−1^] (*K*_aff_=1/*K*_D_)	2.41 × 10^6^
Sensitivity at low *c* [displacement/*M*] (Sensitivity at low *c* = ∆displacement/*K*_D_)	2.73 × 10^8^
LOD [*M*] (3*standard deviation of blank / sensitivity at low *c*)	3.29 × 10^-8^
Detection range [*M*]	1.01 × 10^−7^–6.82 × 10^−7^

## Conclusion

The possibility to prepare biological molecularly imprinted nanoparticles, starting from SilMA, a biocompatible and biodegradable protein extracted from silk, was recently demonstrated [21]. Here, we improved further the knowledge on the process of forming bioMIPs, by testing the effect of smaller templates for the SilMA imprinting; thus, we choose the structured peptide hepcidin as the template. The hepcidin-imprinted bioMIPs proved to be selective for hepcidin and to possess affinity in the high nanomolar range for their template. So far, our results indicate that peptides can indeed be imprinted by using silk fibroin as the macromolecular monomer. The affinity of the bioMIP for the peptide appeared to be lower with respect to that reported when a whole protein is being imprinted, possibly suggesting that the smaller the template, the fewer the interactions between the template and the macromonomer. Relatively to the number of binding sites per bioMIP particle, these appeared to double, from about 1 to about 2 per bioMIP, for the template being a peptide with respect to when the template was a protein. This suggested that the size of the template is inversely proportional to the formed binding sites. Additionally, we showed that bioMIPs were suitable to detect their peptide template even amid the complexity of serum samples. Despite encouraging, the performance of the competitive hepcidin assay should be further tuned to fulfill clinical purposes. It is expected that an improved affinity of the bioMIP for hepcidin would be necessary to allow low nanomolar detection levels. This issue can possibly be addressed by exploring the preparation of bioMIPs by means of solid phase synthetic approaches [4, 5] that are meant to form and enrich MIP nanoparticles onto an affinity column, so to pre-select the nanoparticle population on the basis of homogeneous and high affinity binding sites.

Finally, the driving force to explore protein-derived materials, in particular SilMA, for making MIPs is an effort to broaden the current spectrum of the materials available (and used) for imprinting. Some non-conventional materials are currently under investigation [39], so to widen the boundaries of MIP research and applications. In the present case, silk fibroin bioMIPs offer some interesting advantages with respect to the state-of-the-art formulations commonly used to synthesize the nanoMIPs. Silk fibroin, hence bioMIPs, possesses remarkable biocompatibility and biodegradability, and has a low environmental impact, opening to the fabrication of bioMIPs from industrial wastes. Additionally, silk fibroin degradation byproducts are non-toxic, leading to greener and low polluting MIP generation.

## Supplementary information

ESM 1 (DOCX 68.6 kb)
