# Supplementary material for: BioMIPs: molecularly imprinted silk fibroin nanoparticles to recognize the iron regulating hormone hepcidin
Source: Mikrochim Acta. 2022 Jan 21;189(2):66. doi: 10.1007/s00604-022-05165-0 (PMC8782820; doi:10.1007/s00604-022-05165-0)
Supplement: Supplementary file 1 — (DOCX 68.6 kb) [file 604_2022_5165_MOESM1_ESM.docx]

**Electronic Supplementary Material**

**BioMIPs: molecularly imprinted silk fibroin nanoparticles to recognize the iron regulating hormone hepcidin**

**Alessandra Maria Bossi,^1*^ Devid Maniglio^2^**

1. University of Verona, Department of Biotechnology, Strada Le Grazie 15, 37134 Verona, Italy

2. University of Trento, Department of Industrial Engineering, BIOtech Research Center, Via delle Regole 101, Mattarello, 38123 Trento, Italy

* Corresponding author:

Alessandra Maria Bossi, University of Verona, Department of Biotechnology, Strada Le Grazie 15, 37134 Verona, Italy; phone: +39 045 8027946; fax: +39 045 8027929; email: [alessandramaria.bossi@univr.it](mailto:alessandramaria.bossi@univr.it)

SI1. Estimation of the bioMIP molecular weight by static light scattering

Figure SI 1 Molecular weight (MW) plot. MW is estimated as the intercept of 1/Da.

SI2. Calibration curve for hepfitc.


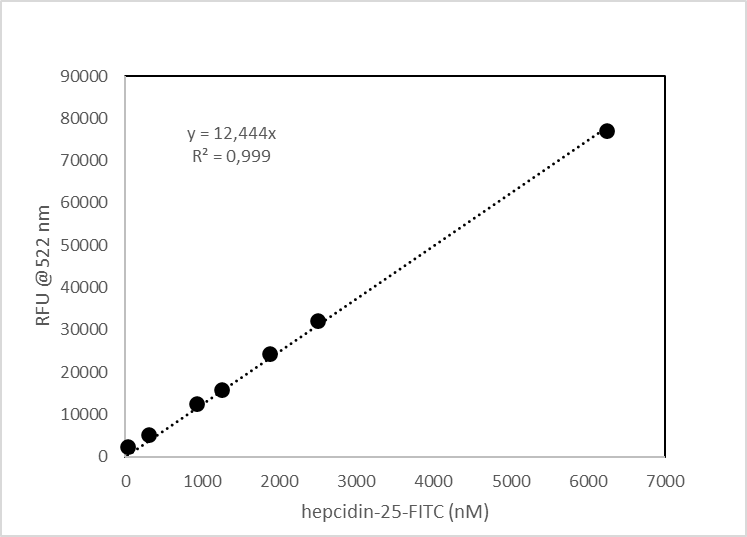


Figure SI 2. Calibration curve for hepfitc. Measures were in triplicate. Stdv5%.
